# Cryo-EM structure of the nonameric CsgG-CsgF complex and its implications for controlling curli biogenesis in Enterobacteriaceae

**DOI:** 10.1371/journal.pbio.3000748

**Published:** 2020-06-19

**Authors:** Manfeng Zhang, Huigang Shi, Xuemei Zhang, Xinzheng Zhang, Yihua Huang

**Affiliations:** 1 Shenzhen Branch, Guangdong Laboratory for Lingnan Modern Agriculture, Genome Analysis Laboratory of the Ministry of Agriculture, Agricultural Genomics Institute at Shenzhen, Chinese Academy of Agricultural Sciences, Shenzhen, China; 2 National Laboratory of Biomacromolecules, CAS Center for Excellence in Biomacromolecules, Institute of Biophysics, Chinese Academy of Sciences, Chaoyang District, Beijing, China; 3 University of Chinese Academy of Sciences, Beijing, China; Technion Israel Institute of Technology, ISRAEL

## Abstract

Curli play critical roles in biofilm formation, host cell adhesion, and colonization of inert surfaces in many Enterobacteriaceae. In *Escherichia coli*, curli biogenesis requires 7 curli-specific gene (*csg*) products—CsgA through G—working in concert. Of them, CsgG and CsgF are 2 outer membrane (OM)-localized components that consists of the core apparatus for secretion and assembly of curli structural subunits, CsgB and CsgA. Here, we report the cryogenic electron microscopy (cryo-EM) structure of CsgG in complex with CsgF from *E*. *coli*. The structure reveals that CsgF forms a stable complex with CsgG via a 1:1 stoichiometry by lining the upper lumen of the nonameric CsgG channel via its N-terminal 27 residues, forming a funnel-like entity plugged in the CsgG channel and creating a unique secretion channel with 2 constriction regions, consistent with the recently reported structure of the CsgG-CsgF complex. Functional studies indicate that export of CsgF to the cell surface requires the CsgG channel, and CsgF not only functions as an adaptor that bridges CsgB with CsgG but also may play important roles in controlling the rates of translocation and/or polymerization for curli structural subunits. Importantly, we found that a series of CsgF-derived peptides are able to efficiently inhibit curli production to *E*. *coli* when administrated exogenously, highlighting a potential strategy to interfere biofilm formation in *E*. *coli* strains.

## Abstract 2

Curli are functional amyloids that play critical roles in biofilm formation and adhesion in many Enterobacteriaceae. Two outer membrane proteins, CsgG and CsgF, form the core apparatus for secretion and assembly of the curli structural subunits. The cryo-EM structure of the CsgG-CsgF complex reveals a unique amyloid secretion channel with two tandem constriction regions.

## Introduction

Curli, a class of “functional” amyloids, are the major proteinaceous component of a complex extracellular matrix produced by many Enterobacteriaceae such as *E*. *coli* and *Salmonella spp*. [1–4]. In contrast to pathogenic amyloids that are associated with several devastating neurodegenerative diseases [5–7], bacterial curli fibers are produced via a highly coordinated biosynthetic process involving many important physiological processes, including cell adherence, invasion, host colonization, and biofilm formation [1,4,8–10].

The assembly of curli subunits into fibers in vivo proceeds via a mechanism known as the nucleation-precipitation pathway (also termed the type VIII bacterial secretion system) [2,11–13]. In *E*. *coli*, at least 7 proteins are dedicated to the structure and biogenesis of curli fibers [2,14]. These proteins are encoded by 2 divergently transcribed curli-specific gene (*csg*) operons, *csgBAC* and *csgDEFG* [15]. Of the 7 *csg* products, CsgA and CsgB are the two structural subunits of curli fibers. CsgA, the major structural subunit, forms the hair-like filaments through orderly polymerization, but its efficient polymerization necessitates the minor structural subunit, CsgB, that acts as a nucleator [16,17]. By contrast, CsgC through G proteins are 5 nonstructural components of curli fibers yet perform diverse functions in curli biogenesis: CsgC is a chaperone-like protein that prevents curli subunits from premature polymerization in the periplasm [18,19]; CsgD, a master regulator of the *csgBAC* operon, is responsive to many environmental cues, coordinating the timely expression of the *csgBAC* operon [14,20]; the periplasmic accessory protein CsgE is believed to carry CsgA across the periplasm to the outer membrane (OM) prepared for curli subunit secretion by the OM-localized CsgG channel [2,21,22]; and the extracellular accessory protein CsgF, along with CsgB, is critical for CsgA fiber elongation and attachment to the OM, making curli a major type of surface organelles in gram-negative bacteria [15,23]. The assembled curli fibers exhibit typical biophysical and biochemical properties of amyloids, which are characterized by the presence of cross β-strand structures that bind to the dyes Congo red (CR) and thioflavin T [2,14,16,24].

A recent remarkable progress in the field is the structural determination of the curli secretion channel, CsgG [25,26]. The structures reveal that the lipoprotein CsgG forms a nonameric 36-stranded β-barrel secretion channel with a constriction region located in the middle. The eyelet of constriction region is approximately 12 Å in diameter, suggesting that curli subunits are secreted across the OM in an unfolded manner. Further cryogenic electron microscopy (cryo-EM) maps of the CsgG-CsgE complex indicate that CsgE could oligomerize to form nonamers in the periplasm, “capping” the periplasmic side of the CsgG channel [26,27]. To date, the structures of CsgC [18], CsgE [26,28], CsgF [29], and the CsgG-CsgF complex [30] are also revealed. Despite the availability of these isolated structures and the established functional roles of these individual *csg* proteins in curli biogenesis, the transport details of curli subunits, the overall architecture of the curli secretion apparatus, and ways to interfere with curli biogenesis await further elucidation.

In this work, we show that CsgF forms a stable complex with CsgG via a 1:1 stoichiometry by lining the extracellularly faced lumen of the nonameric CsgG channel via its N-terminal 27 residues, creating a secretion channel with 2 constriction regions for curli subunit secretion. Functional studies indicate that export of the accessory protein CsgF to the cell surface requires CsgG, and several lumen-facing residues of CsgF in the nonameric CsgG-CsgF channel play important roles in curli production. Furthermore, we found that a series of CsgF-derived peptides are able to efficiently inhibit curli production of both the wild-type (WT)- and the Δ*csgF*-*E*. *coli* K-12 BW25113 strains when administrated exogenously, highlighting a potential strategy to interfere with biofilm formation in *E*. *coli* strains.

## Results

### Overall architecture of the nonameric CsgG-CsgF complex

**Fig 1:**
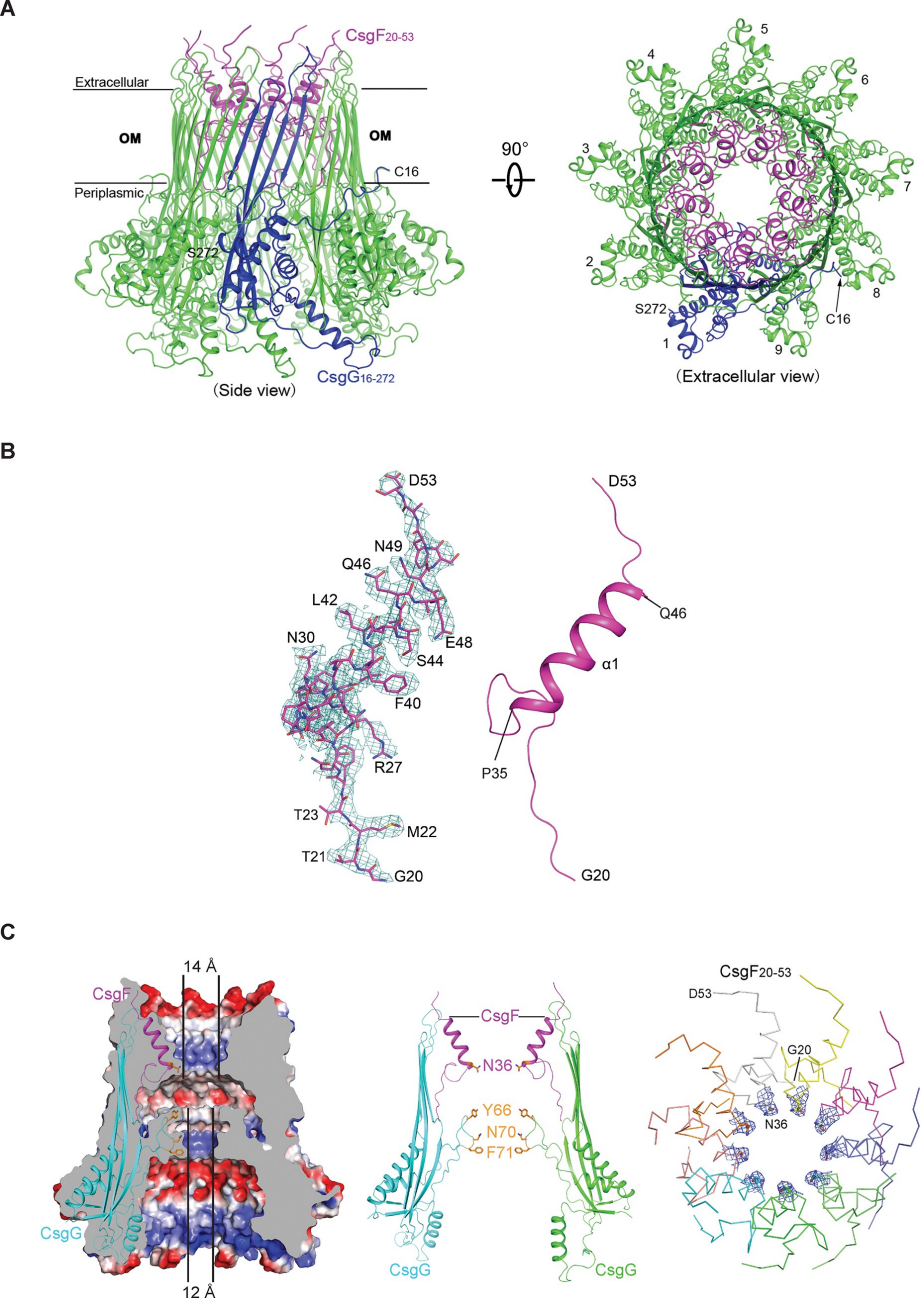
Overall structure of the CsgG-CsgF complex. (A) Overall structure of the nonameric CsgG-CsgF complex shown in ribbon representation from different perspectives: side view (left) and extracellular view (right). The CsgG-CsgF complex contains 9 CsgG-CsgF subcomplexes. CsgG (one CsgG monomer is highlighted in blue) and CsgF are colored in green and violet, respectively. In the complex structure, the C-terminal 5 residues of CsgG and 85 residues of CsgF are invisible. (B) Structure of the CsgG-bound CsgF_20–53_ fragment. Cryo-EM densities of CsgF_20–53_ fragment are shown on the left (blue), and its secondary structures in ribbon are shown on the right (violet). (C) Close-up view of the constriction regions of the CsgG-CsgF channel. Slab view of the electrostatic surface representation of the CsgG-CsgF channel showing the 2 constriction regions (left) and the lining residues at the 2 eyelets (middle). The CsgG constriction region and the CsgF constriction region have pore dimensions of approximately 12 Å and 14 Å, respectively, in diameter (shortest atom-to-atom distance). Map densities and side chains of the 9 Asn36 of CsgF that consist of the CsgF constriction region are shown on the right. Csg, curli-specific gene product; OM, outer membrane.

To obtain the structure of the CsgG-CsgF complex, the full-length *E*. *coli csgG* and *csgF* genes were co-expressed in *E*. *coli* strain BL21 (DE3), and the purified CsgG-CsgF complex was applied to the glow-discharged GIG R1/1(Cu) grid for cryo-EM specimen preparation. We collected cryo-EM images for the sample, and cryo-EM structure determination using multiple rounds of three-dimensional (3D) classification and refinement resulted in a density map with an overall resolution of 3.6 Å (S1A, S1B, S1C and S1D Fig). The map/model Fourier shell correction (FSC) curves and local resolution estimations are consistent with the gold-standard resolutions from FSC curves between half maps of the split data (S1E Fig). Atomic models were built and refined against the map (S1F Fig) using the crystal structure of the nonameric CsgG as initial model (Protein Data Bank [PDB] code: 3X2R) [25], and the extra densities in the resulted map corresponding to CsgF were manually built according to its sequence, and refined. Overall, the structure of the CsgG-CsgF complex reveals that CsgF forms a 1:1 stable complex with CsgG via its N-terminus (residues 20–53 of the full-length CsgF), yet its C-terminus including residues 54–138 is invisible in the map due to flexibility (Fig 1A and S1B Fig). The nonameric CsgG structure in the CsgG-CsgF complex is almost identical to the crystal structure of the nonameric CsgG with a root-mean-square deviation (RMSD) of approximately 0.5 Å for all 1,854 aligned Cα atoms. By contrast, the visible CsgG-bound CsgF fragment adopts a loop-helix-loop conformation (Fig 1B), distinct from its nuclear magnetic resonance (NMR) structure but consistent with the result of secondary structural prediction [29] (S2A and S2B Fig, S1 Data). Strikingly, the nonameric CsgG-CsgF complex forms a secretion pore with 2 constriction regions (Fig 1C). In addition to the constriction region that is lined with 3 stacked 9-residue rings consisting of either Tyr66, Asn70, or Phe71 of CsgG [25,26], the CsgG-bound CsgF adds another constriction region to the CsgG channel. This pore eyelet is formed by 9 Asn36 residues, contributed by each CsgF molecule, with a diameter of approximately 14.8 Å (Fig 1C). Overall, the structure of the CsgG-CsgF complex (CsgG includes residues 16–272 and CsgF includes residues 20–53) is almost identical to that of the recently reported CsgG-CsgF complex (CsgG includes residues 25–277 and CsgF includes residues 20–55, PDB code: 6L7A) [30], with an RMSD of 0.8 Å for all 2,538 aligned Cα atoms.

To improve the resolution of the cryo-EM structure of the CsgG-CsgF complex in order to accurately locate side chains of 9 Asn36 residues of CsgF that consist of the upper constriction region of the CsgG-CsgF channel, the purified CsgG-CsgF complex was proteolytically digested with chymotrypsin to remove flexible regions of the CsgG-CsgF complex that may affect the stability of the complex in the detergent-containing buffers and increase particle heterogeneity on the grid. The resulted protein sample (denoted as the CsgG-CsgF_20–59_ complex) contained a variant of CsgG that includes residues 47–252 in complex with the most N-terminal 40 residues of the mature CsgF (CsgF_20–59_), as confirmed by mass spectrometric analysis (S3 Fig). In contrast to the oligomerization state exhibited on size exclusion chromatography (S3A Fig), the CsgG-CsgF_20–59_ complexes tended to form filaments with varied lengths on the cryo-EM grids (S4A and S4B Fig). Using a similar cryo-EM structure determination approach as described earlier, we obtained 2 maps for the nonameric CsgG-CsgF_20–53_ complex and the nonameric CsgG-CsgF_20–59_ complex, with an overall resolution of 2.9 Å and 3.2 Å, respectively (S4C, S4D, S4E and S4F Fig). Intriguingly, there exist 2 types of non-physiological inter-complex interactions: a head-to-head homodimeric interaction and a head-to-tail heterodimeric interaction within the filaments (S5A and S5B Fig). The structure of the nonameric CsgG-CsgF_20–59_ complex was obtained from an interacting mode in the filaments through which the most C-terminal 6 residues of each CsgF_20–59_ fragment interact with the periplasmic regions of each CsgG, stabilizing the conformation of the 6-residue loop of CsgF_20–59_ in the structure but with relatively weak densities (S5C Fig). Structural analysis indicated that formation of the filaments of the CsgG-CsgF_20–59_ complex on the grids during cryo-EM sample preparation may result from 2 types of inter-complex charge-charge interactions (S5A and S5B Fig). To test this, we found that increased salt concentrations in the sample buffer significantly decreased the lengths of the filaments formed on the grids (S5D Fig). As expected, the 2.9-Å cryo-EM map of the CsgG-CsgF_20–53_ complex allows us to ascertain all the side chains of CsgF_20–53_ unambiguously—in particular side chains of the 9 Asn36 residues that consist of the upper constriction region of the CsgG-CsgF channel (Fig 1C, right). Taken together, CsgG and CsgF form a stable nonameric complex via 1:1 stoichiometry, which creates a unique secretion channel with 2 constriction regions, representing the core apparatus of the curli secretion machinery in the OM.

### Interactions between CsgG and CsgF

**Fig 2:**
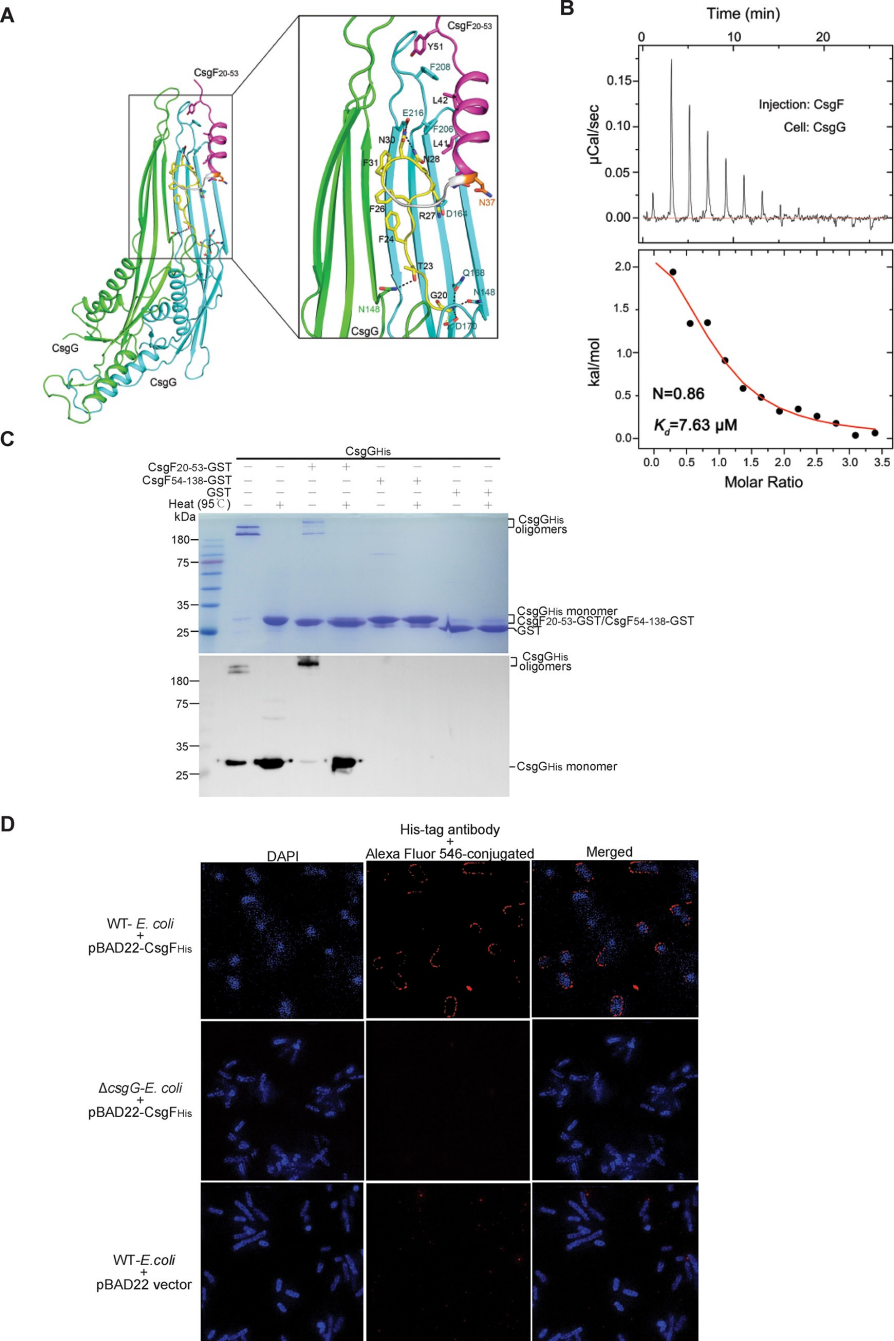
Characterization of the interactions between CsgG and CsgF. (A) Close-up view interactions between CsgG and CsgF in the CsgG-CsgF complex. Two adjacent CsgG molecules are colored in green and cyan. The most N-terminal loop (including residues 20–31, in yellow) of CsgF primarily binds at the joint area of 2 adjacent CsgG monomers; the α helix (including residues 37–46, in violet) of CsgF interacts with one CsgG monomer, and the connecting loop (including residues 32–36, in grey) of CsgF has no interaction with CsgG. (B) ITC measurement of the affinity between CsgG and the full-length CsgF. The underlying numerical data for the figure are shown in S1 Data. (C) GST pull-down experiments (upper panel) and western blot to detect CsgG_His_ (bottom panel). CsgG nonamers and the nonameric CsgG-CsgF_20–53_ complexes were unable to be dissociated to monomers on the 12% SDS-PAGE unless the samples were boiled at 95°C for 10 minutes before loading. CsgF_20–53_-GST has a close molecular mass (approximately 28.9 kDa) to CsgG_His_. (D) Immunofluorescence assays showing that surface exposure of CsgF requires CsgG. DAPI is a fluorescent stain that binds strongly to adenine–thymine rich regions in DNA to show the location of the bacterial cells. Csg, curli-specific gene product; DAPI, 4′,6-diamidino-2-phenylindole; GST, glutathione s-transferase; ITC, isothermal titration calorimetry; WT, wild-type.

Despite an overall 1:1 stoichiometry between CsgG and CsgF in the nonameric CsgG-CsgF complex, each CsgF molecule binds 2 adjacent CsgG monomers in the CsgG nonameric channel (Fig 2A). Specifically, the most N-terminal 12 residues of the full-length CsgF (residues 20–31), which adopt a loop conformation, line the inner surface of the upper lumen of the nonameric CsgG channel. Residues 36–46 of CsgF fold as a helix that leans on the inner wall of the upper lumen, and all 9 helices together form a funnel-like entity plugged in the upper lumen of the CsgG channel; following the helix is a short loop consisting of residues 47–53 of CsgF that extends out extracellularly. The interactions between CsgG and CsgF involve polar interactions, van der Waals contacts, and hydrophobic interactions (Fig 2A). Notably, the amino group of Gly20, the first residue of the mature CsgF, hydrogen-bonds to the side chains of residues Asn148, Gln168, and Asp170 of CsgG; hydrophobic interactions are found between residues Leu41 and Leu42 of CsgF and residues Phe206 and Phe208 of CsgG. Residues Phe24, Phe26 and Phe31of CsgF are in a van der Waals contact distance to a cluster of lumen-facing residues from the adjacent CsgG molecule. In summary, there are 2 major features of the CsgG-CsgF interaction. First, the N-terminus of CsgF binds to CsgG in a bipartite mode, with a connecting loop (residues G32–P35) that extends to the center of the CsgG channel to separate the 2 binding motifs of CsgF. Second, CsgF binds 2 adjacent CsgG molecules simultaneously, which may enhance the overall stability of the nonameric CsgG-CsgF channel, completing the unique secretion apparatus dedicated to secretion and polymerization of curli subunits.

As the NMR structure of the N-terminus (residues 20–59) in the isolated CsgF adopts a remarkably different conformation to that of the CsgG-bound state [29] (Fig 1B and S2A Fig), we want to know whether the purified recombinant CsgF is able to bind the nonameric CsgG channel. To test this, we performed isothermal titration calorimetry (ITC) to characterize the interaction between CsgG and CsgF. As shown in Fig 2B (S1 Data), the binding affinity between the 2 full-length proteins is approximately 7.6 μM (*Kd* = 7.63 μM) with a close molar ratio of 1:1 (N = 0.86). A combination of the ITC and the cryo-EM results suggest that binding to CsgG may induce conformational changes of the N-terminus of CsgF, allowing its insertion into the CsgG channel. Because the C-terminus of CsgF is invisible in our structures, we want to further know whether the C-terminus of CsgF also contributes to the CsgG-CsgF interaction. To examine this, we performed glutathione s-transferase (GST) pull-down experiments followed by western blot confirmation using recombinant CsgF variant proteins that are fused to GST C-terminally. Consistent with our structural observation that only the N-terminus of the mature CsgF (residues 20–53) binds CsgG, CsgF_20–53_-GST—but neither CsgF_54-138_-GST nor GST alone—pulled-down C-terminally His-tagged CsgG (Fig 2C). Furthermore, as the structure of the CsgG-CsgF complex shows that CsgF is a surface-exposed protein, we ask whether its surface exposure requires CsgG, i.e., whether the accessory component, CsgF—like curli structural subunits CsgA and CsgB—is also secreted to the cell surface via the CsgG channel. To clarify this, we used the Δ*csgG*-*E*. *coli* K-12 BW25113 strain [25] and carried out immunofluorescence assays. First, the full-length *csgF* gene with a C-terminal 6×His tag coding sequence was cloned into pBAD22 vector. The constructed plasmid pBAD22-CsgF_His_ was transformed into the WT-*E*. *coli* K-12 BW25113 cells, and then induced. We incubated the resultant *E*. *coli* cells with primary anti-His antibody, followed by Alexa Fluor 546-conjugated (red-fluorescing) secondary antibody for fluorescence detection. Clearly, *E*. *coli* cells that harbored CsgF_His_-expressing plasmids displayed red fluorescence circles, suggesting that the C-termini of CsgF_His_ are surface exposed (Fig 2D, upper panels). Under the same scenarios, no red fluorescence was observed for either the Δ*csgG*-*E*. *coli* K-12 BW25113 cells that harbored pBAD22-CsgF_His_ plasmids or the WT-*E*. *coli* cells transformed with a vacant pBAD22 vector (Fig 2D). Taken together, our immunofluorescence assays confirmed that CsgF is a surface-exposed protein and its surface localization requires the CsgG channel.

### The lumen-facing residues of CsgF in the nonameric CsgG-CsgF channel affect curli production

**Fig 3:**
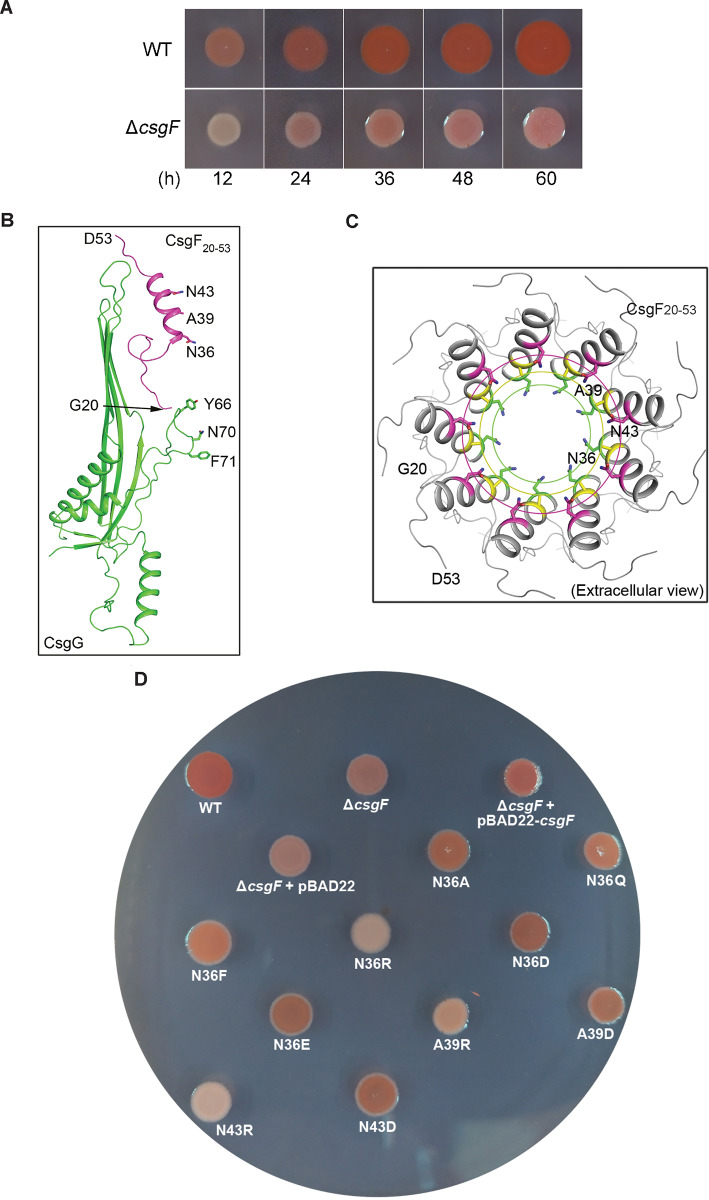
Mutational analysis of the three lumen-facing residues of CsgF. (A) CR staining assays showing curli production of the WT- and the Δ*csgF*-*E*. *coli* K-12 BW25113 strains over time. Pictures were taken at 5 different incubation time points (12, 24, 36, 48, and 60 hours, respectively). (B) Positions of CsgF residues selected for mutational studies in the CsgG-CsgF complex. (C) Residues of CsgF (N36, A39, and N43) point to the lumen of the nonameric CsgG-CsgF channel (extracellular view). Residues are shown in stick mode. (D) Complementary assay using CR staining. Mutants N36R, A39R, and N43R displayed significantly reduced curli production. CR, Congo red; Csg, curli-specific gene product; WT, wild-type.

Our previous mutational analysis of the CsgG secretion channel indicated that a positively charged upper luminal environment disfavors curli production [25]. To assess the functional roles of potentially critical residues of CsgF in curli production, the Δ*csgF*-*E*. *coli* K-12 BW25113 strain was constructed and then transformed with CsgF variants prior to assessment of curli production. Unlike the Δ*csgG*-*E*. *coli* K-12 BW25113 strain that prohibits curli production by blocking secretion of curli structural subunits and exhibits a strikingly “white” phenotype even over a 60-hour incubation time, as determined by using CR staining assay [22,25], the Δ*csgF*-*E*. *coli* K-12 BW25113 strain progressively turned red over the incubation time [14] (Fig 3A). This phenomenon indicates that deletion of the *csgF* gene may not prevent secretion of curli structural subunits in the Δ*csgF*-*E*. *coli* K-12 BW25113 strain but may only cause a decreased translocation rate and/or polymerization rate of curli structural subunits. Using the CR staining assay, we further analyzed several sets of CsgF mutants for their ability to restore curli production.

We first studied the effects of point mutations that are localized in the constriction region formed by Asn36 of CsgF on curli production (Fig 3B and 3C). Consistent with a previous report, close to the WT levels of curli production were restored upon induction of *csgF* gene expression from the transformed plasmid in the Δ*csgF*-*E*. *coli* K-12 BW25113 strain [23] (Fig 3D). When mutated to 6 different types of residues (Ala, Phe, Gln, Glu, Asp, or Arg), only Asn36Arg mutant exhibited strikingly decreased curli production (Fig 3D) [30]. These findings also imply that the slight change of dimension of this secretion pore might not be a key factor in affecting curli subunit secretion, but a relatively positively charged microenvironment at this region disfavors curli production. In line with this view, mutants Ala39Arg and Asn43Arg, two other lumen-facing residues in the nonameric CsgG-CsgF channel (Fig 3B and 3C), also exhibited remarkably reduced curli production levels (Fig 3D). These observations agree with our previous studies showing that a positively charged microenvironment in the upper lumen of the isolated CsgG channel affected curli production [25]. Taken together, our complementation assays implied not only that CsgF simply bridges the nucleator CsgB with the CsgG channel by serving as an adaptor but also that its lumen-facing residues also play important roles in controlling the rates of translocation and/or polymerization of curli structural subunits by providing an optimal microenvironment for efficient curli production.

### The CsgF-derived peptides inhibit curli biogenesis

**Fig 4:**
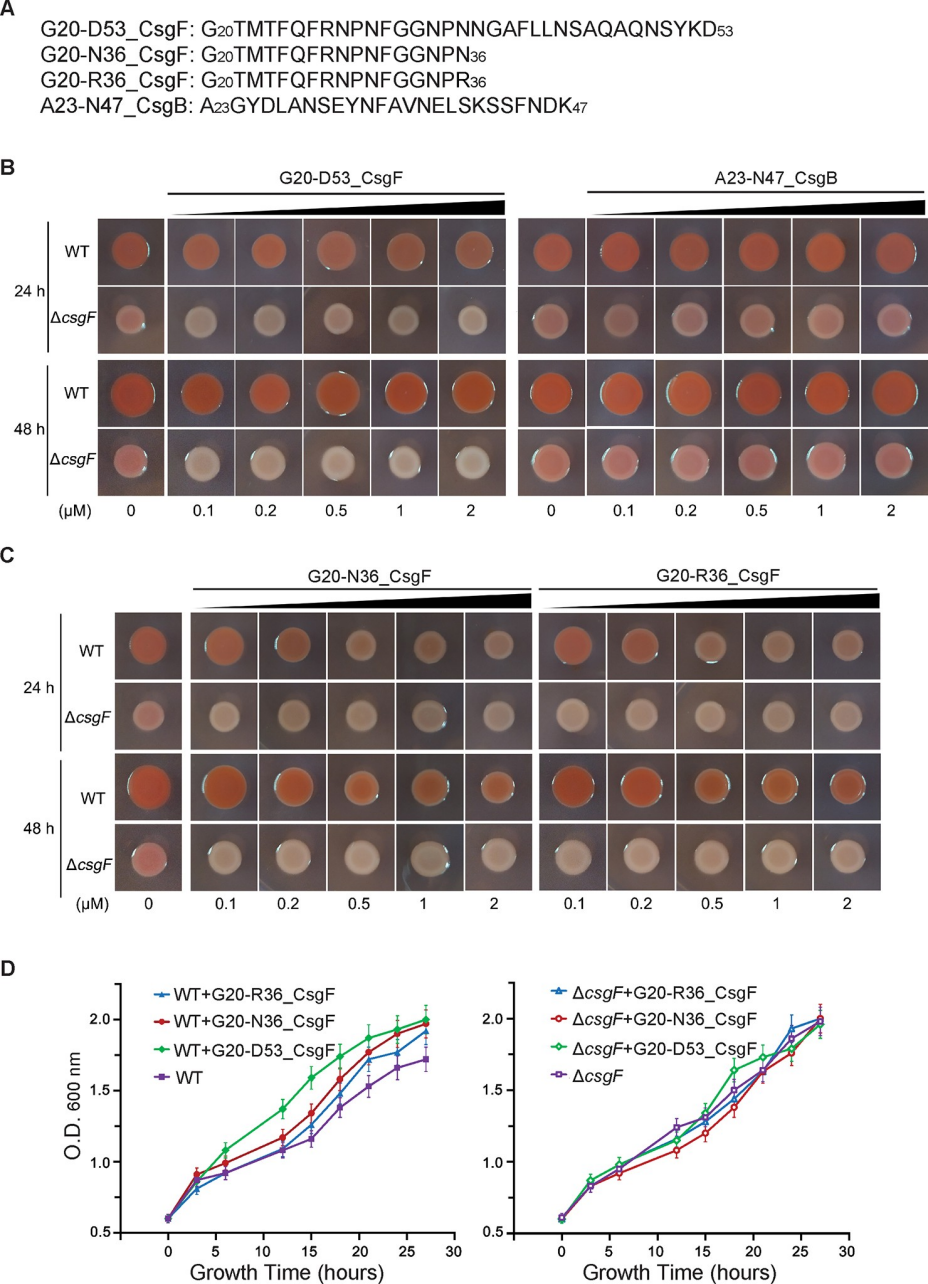
The CsgF-derived peptides inhibit curli production. (A) Amino acid sequences of 4 synthesized peptides. (B) Effects of peptides G20-D53_CsgF (left) and A23-N47_CsgB (right) on curli production of both the WT- and the Δ*csgF*-*E*. *coli* strains. (C) Effects of peptides G20-N36_CsgF (left) and G20-R36_CsgF (right) on curli production of both the WT- and the Δ*csgF*-*E*. *coli* strains. (D) Cell growth curves of the WT-*E*. *coli* (left) and the Δ*csgF*-*E*. *coli* (right) stains in presence or absence of 3 CsgF-derived peptides. The underlying numerical data for the figure can be found in S1 Data. Csg, curli-specific gene product; O.D., optical density; WT, wild-type.

Curli biogenesis is critical for biofilm development in enterobacterial strains [1,4,31,32]. Our structure of the CsgG-CsgF complex and in vitro binding assay show that the N-terminal fragment of CsgF directly binds CsgG. This prompts us to further investigate whether synthesized CsgF-derived peptides based on the structure of the CsgG-CsgF complex are able to inhibit curli biogenesis when administrated exogenously. To this end, we first synthesized 2 different lengths of CsgF-derived peptides (G20-D53_CsgF and G20-N36_CsgF, containing 34 and 17 residues of the most N-terminus of the mature CsgF, respectively) and 1 CsgB-derived peptide (A23-N47_CsgB, containing the most N-terminal 25 residues of the mature CsgB) (Fig 4A) and tested their effects on curli production of both the WT- and the Δ*csgF*-*E*. *coli* K-12 BW25113 strains. Clearly, G20-D53_CsgF had strikingly inhibitory effects on curli production to the Δ*csgF*-*E*. *coli* K-12 BW25113 strain even at low concentrations in an incubation time of 48 hours, yet little effect to the WT *E*. *coli* strain was achieved (Fig 4B). However, synthesized peptide A23-N47_CsgB exhibited no inhibitory effects on curli production to either the WT- or the Δ*csgF*-*E*. *coli* K-12 BW25113 strains (Fig 4B, right), suggesting that the inhibitory effects on curli production of the Δ*csgF*-*E*. *coli* K-12 BW25113 strain achieved by G20-D53_CsgF is specific. Importantly, G20-N36_CsgF, a relatively shorter peptide derived from the N-terminus of CsgF, had a pronounced inhibitory effect on curli production of both the WT- and the Δ*csgF*-*E*. *coli* K-12 BW25113 strain at sub-micromolar concentrations (Fig 4C, left), in line with a previous observation [30]. Since our previous complementary assays indicated that Asn36Arg mutant exhibited significantly decreased curli production (Fig 3D), we further synthesized a peptide G20-R36_CsgF in which the last residue Asn36 was replaced by Arg and tested its effects on curli production. As shown in Fig 4C (right), G20-R36_CsgF had a similar, but not a more pronounced, inhibitory effect on curli production of both the WT- and the Δ*csgF*-*E*. *coli* K-12 BW25113 strains. To rule out the possibility that suppression of curli production by these CsgF-derived peptides resulted from their effects on bacterial growth or metabolism, we performed cell growth assays in presence or absence of the above-tested peptides. Apparently, none of these CsgF-derived peptides showed cytotoxicity to either the WT- or the Δ*csgF*-*E*. *coli* K-12 BW25113 strains (Fig 4D, S1 Data). In summary, all 3 CsgF-derived peptides have pronounced inhibitory effects on curli production of the Δ*csgF*-*E*. *coli* K-12 BW25113 strain, but only peptides G20-N36_CsgF and G20-R36_CsgF are able to efficiently inhibit curli production of the WT-*E*. *coli* strain. These findings indicate that the peptide G20-N36_CsgF could serve as a template for further designing small chemicals with enhanced affinity for inhibiting curli production to attenuate biofilm formation in the WT-*E*. *coli*.

## Discussion

Understanding the molecular mechanisms underlying curli biogenesis is important for developing therapeutics to attenuate biofilm formation in enterobacteria and potentially pathogenic amyloidogenesis in humans [1,31,33,34]. CsgG and CsgF are the 2 components of the core machinery in the OM for curli assembly. The structure of the CsgG-CsgF complex demonstrates that CsgF and CsgG form a stable complex at a molar ratio of 1:1, revealing a unique secretion channel with 2 distinct restriction regions along the curli subunit translocation pathway, consistent with a recent report by Yan and colleagues [30]. From the structure of the CsgG-CsgF complex and the isolated CsgF NMR structure [29], we learned that the orientation between the N-terminus and the C-terminal domain of CsgF is not conformationally fixed, which made the C-terminus of CsgF invisible in the cryo-EM structure. As CsgB is able to bind CsgF [30], how many copies of CsgB bind the nonamic CsgG-CsgF complex, how they mutually interact, and whether binding of CsgB to CsgF is able to stabilize the overall conformation of CsgF are interesting questions to answer in the field.

The CsgG-CsgF complex structure, combined with functional analysis, also provides important clues to understanding how curli is orderly assembled and attached to the cell surface. Apparently, for efficient assembly of a single curli fiber, individual CsgF molecules must be first translocated to the cell surface via the CsgG channel and stick to the upper lumen via its N-terminus one after another, forming the integral core secretion apparatus for subsequent secretion of curli structural subunits, CsgB and CsgA. The free C-terminal domain of CsgF recruits and binds the secreted nucleator CsgB, creating a growth point for further addition of CsgA subunits [23]. In agreement with this process, the Δ*csgF*- and Δ*csgB*-*E*. *coli* strains display similar defects on curli production [16,23]. In other words, both the Δ*csgF*- and the Δ*csgB*-*E*. *coli* strains progressively turn red in CR staining assays over the incubation time, arguing that deletion of the *csgF* or *csgB* genes should not abolish secretion of curli structural subunits, and the secreted curli structural subunits will polymerize to form filaments extracellularly, albeit less efficiently. Our complementation assays also highlight that CsgF plays a number of roles during curli biogenesis. In addition to functioning as an adaptor that bridges the CsgB being secreted with the CsgG secretion channel, it may also provide an optimal chemical microenvironment for protein folding as well as create a spatial proximity for CsgA to access CsgB, rendering an efficient curli assembly process in bacteria. In particular, in an energy-free environment, the translocation rates of the curli structural subunits should highly depend on the folding rates of the subunits being transported. In this regard, the nature of the microenvironment in the upper lumen of the CsgG-CsgF channel may be important for facilitating the folding of CsgB, which, in turn, affects the translocation rates for both CsgB and CsgA across the OM.

The structure CsgG-CsgF complex may provide clues for designing CsgF-derived peptides for controlling biofilm formation in *E*. *coli* strains. In general, our CR staining assays show that all the CsgF-derived peptides had a much more striking inhibitory effect on the Δ*csgF*-*E*. *coli* K-12 BW25113 strain than the WT-*E*. *coli* K-12 BW25113 strain. The resultant discrepancy is probably due to the fact that the WT-*E*. *coli* is capable of expressing endogenous CsgF, which is able to form some functional CsgG-CsgF channels even in the presence of CsgF-derived peptides. The substantially inhibitory effects—analogous to the Δ*csgG*-*E*.*coli* K-12 BW25113 strain—achieved by all 3 CsgF-derived peptides (G20-N36_CsgF, G20-R36_CsgF, and G20-D53_CsgF) on curli production of the Δ*csgF*-*E*. *coli* K-12 BW25113 strain suggest that binding of these peptides to the CsgG channel may block the secretion of curli structural subunits. An examination of the amino acid sequences of 3 peptides reveals that they all contain the connecting loop residues that may be able to reach the position of the upper constriction region of the CsgG-CsgF channel (Fig 2A). We speculate that binding of these peptides to the CsgG channel, residues that consist of the connecting loop of CsgF, might not adopt the same conformation as what is observed in the CsgG-CsgF structures and therefore are unable to create a functional secretion pore for subsequent secretion of CsgB and CsgA. This is possible because the C-termini of the peptides G20-N36_CsgF and G20-R36_CsgF are not fixed by the other CsgG-binding motif of CsgF (the leaned α helix, CsgF36-D53), while the longest peptide G20-D53_CsgF, which contains 2 separate CsgG-binding motifs, might cause incorrect binding to the CsgG channel in vitro. Given that the export of CsgF via the CsgG channel may proceed one after another in vivo, this could allow orderly and correct folding of CsgF fragments. Whereas these peptides access the binding sites on the nonameric CsgG simultaneously in vitro, it is likely that the upper constriction region is not functionally assembled by these CsgF-derived peptides upon binding to the nonameric CsgG channel. In particular, the upper constriction region formed by 9 Asn36 of CsgF only has a pore dimension of 14.8 Å in diameter; incorrect folding and arrangement of the connecting loop might readily cause the blockage of the secretion of curli structural subunits. Consistent with this speculation, synthesized peptide G20-F31_CsgF, which does not contain the connecting loop residues but presumably has the same affinity as that of G20-N36_CsgF and G20-R36_CsgF to the CsgG channel, did not show any inhibitory effects on curli production to either the WT- or the Δ*csgF*-*E coli* K-12 BW25113 strains, nor did a shorter peptide G20-R27_CsgF (S6 Fig). Taken together, our current structure and functional studies suggest that the peptide G20-N36_CsgF could serve as an initial template for designing small chemicals to attenuate curli biogenesis and thus potentially control biofilm formation in *E*. *coli* strains.

## Materials and methods

### Cloning, expression, and purification of the CsgG-CsgF complex

The *csgG* and *csgF* genes were isolated from the genomic DNA of the *E*. *coli* strain K12 MG1655 genomic DNA (ATCC) using PCR and were individually cloned into pQLink vector [35] using BamHI and NotI cutting sites, with a 6×His tag attached to the C-terminus of CsgG. The two plasmids were combined step by step by ligation-independent pathway. The pQLink plasmid that co-expresses CsgG and CsgF was transformed into *E*. *coli* strain BL21 (DE3) cells for protein expression. Protein expression was induced by adding 0.4 mM isopropyl β-d-1-thiogalactopyranoside (IPTG) at 26°C overnight. Cells were subsequently harvested by centrifugation at 4,500*g* for 30 minutes at 4°C. Cell pellets were resuspended in 1×PBS, lysed by sonication (Misonix Sonicator S-4000; Cole Parmer, Vernon Hills, IL), and then centrifuged at 39,000*g* for 1 hour at 4°C to collect the total cell membranes. To remove inner membranes, the total membranes were further solubilized for 1 hour at 4°C in 1×PBS containing 1% N-lauroylsarcosine (>95%, Sigma-Aldrich). The OMs were isolated by centrifugation at 39,000g for 1 hour at 4°C, and the resultant membranes were further solubilized 4 hours in 1×PBS containing 40 mM imidazole and 1% LDAO (Anatrace). The supernatants were collected after centrifugation at 39,000*g* for 1 hour at 4°C, and incubated with Ni-NTA agarose beads for 2 hours at 4°C. After rinsing the Ni-NTA agarose beads with wash buffer (1×PBS containing 60 mM imidazole and 0.5% DDM), the CsgG-CsgF complex protein was eluted using 1×PBS containing 250 mM imidazole and 0.1% DDM.

To obtain the CsgG-CsgF_20–59_ complex, the eluted CsgG-CsgF complex was subjected to chymotrypsin proteolysis for 3 hours at room temperature, with an approximate ratio of 100:1 (protein:enzyme). The chymotrypsin-treated protein sample was concentrated to 500 μL and was further applied to size exclusion chromatography on a Superose 6 10/300 GL column (GE Healthcare) that was pre-equilibrated with 20 mM Tris-HCl (pH 8.0), 150 mM NaCl, and 0.06% DDM. The chymotrypsin-treated CsgG-CsgF complex protein eluted as one major peak. Mass spectrometry and N-terminal sequencing confirmed that chymotrypsin removed the N-terminal 31 residues and C-terminal 25 residues of the mature CsgG, as well as C-terminal 78 residues of the mature CsgF.

### Cryo-EM specimen preparation, data acquisition, and processing

The CsgG-CsgF complex (or the CsgG-CsgF_20–59_ complex) was concentrated to approximately 6 mg/mL for cryo-EM specimen preparation. Approximately 3 μL aliquots of protein samples were applied to glow-discharged GIG R1/1(Cu) grid. Grids were flash plunged into liquid ethane at approximately −180°C after being blotted for 4.5 seconds by automatic plunging device EMGP (Leica).

The datasets for the CsgG-CsgF_20–59_ complex were collected in FEI Talos Arctica equipped with direct detector K2 summit (super resolution mode) and GIF quantum energy filter (energy width of 20 eV) at magnifications of 130,000 yielding binned pixel sizes of 1 Å. The corresponding dose rates for 3 datasets were approximately 9.76 e/Å^2^/s, and the exposure times were 5.12 seconds. The images were fractionated into 32 frames. A total of 1,350 micrographs were collected for 3 datasets with the defocus ranging from 1 μm to 2 μm. The beam induced motion was corrected by MotionCor2 [36]. The defocus parameters were estimated by CTFFIND4 [37]; 1,130 motion-corrected micrographs were kept after inspection by eyes. About 49,349 particles were picked by e2boxer.py (a semiautomatic particle picking software) [38] and were processed by 2D classification yielding featured 2D averages. These featured 2D averages were used as references for automatic particle picking from selected micrographs using RELION [39], which resulted in 265,188 particles. After 2 rounds of reference-free 2D classification, 247,227 particles were kept from well-featured classes for further data processing. The initial model for 3D classification was generated from CsgG (PDB code: 3X2R). The 3D classification with C9 symmetry imposed indicates that the complex has head-to-head and head-to-tail intermolecular interactions. For the head-to-head complex, 101,241 particles were selected from one of 7 classes. They were re-centered and re-extracted. The initial refinement of all selected particles from 3D classification with D9 symmetry yielded a 3.1-Å EM map. After per-particle defocus refinement and beam tilt estimation by CTF refinement approach in RELION3.0 [40], 101,241 particles yielded 3D reconstruction with resolutions of 2.9 Å. For the head-to-tail complex, 96,558 particles with C9 symmetry yielded a 3.2-Å reconstruction map.

For building the model of the CsgG-CsgF_20–59_ complex, the crystal structure of the nonameric CsgG (PDB code: 3X2R) was fitted into the cryo-EM map by Chimera [41] and manually adjusted in Coot [42] according to the density. The N-terminus of CsgF was manually built de novo. The model was further refined in real space by PHENIX [43]. Cryo-EM data collection, refinement, and validation statistics are presented in S1 Table. Sample preparation and image acquisition for the CsgG-CsgF complex followed a similar procedure as described earlier for the CsgG-CsgF_20–59_ complex.

### ITC experiment

The ITC experiments were performed using an ITC200 instrument (Microcal Instruments) at 25°C. The CsgG and CsgF protein samples were applied to size exclusion chromatography with a buffer containing 20 mM Tris-HCl (pH 7.5), 150 mM NaCl, and 0.1% (w/v) DDM by gel filtration chromatography. CsgG (20 μM and 250 μL) was in the cell, and CsgF (300 μM and 60 μL) was in the syringe. The titration experiments followed by 12 injections of 3 μL each at an interval of 120 seconds and a stirring rate of 650 rpm. The data were fitted using the Origin 7.0 software package of MicroCal ITC200 implementation.

### GST pull-down assay and western blot

To perform the GST pull-down experiment, we first fused a GST tag C-terminally to fragments of CsgF_20–53_ and CsgF_54–138._ Three plasmids that express CsgF_20–53_-GST, CsgF_54–138_-GST, or GST were individually transformed to the *E*. *coli* BL21 (DE3) strain. The cells were induced with 0.5 mM IPTG at 18°C overnight. Purified protein samples CsgF_20–53_-GST, CsgF_54–138_-GST, or GST (approximately 0.1 mg) were immobilized on glutathione sepharose resin (GE Healthcare). After addition of approximately 0.2 mg of CsgG, the mixtures were incubated for 1 hour at 4°C. The protein-bound resins were washed 3 times with buffer containing 20 mM Tris-HCl (pH 7.5), 150 mM NaCl, 5% (v/v) glycerol, and 0.1% (w/v) DDM to wash unbound or nonspecific bound proteins. The eluted samples were subject to 12% SDS-PAGE analysis.

For western blot, the proteins were then transferred to a PVDF membrane and blocked using TBST buffer (20 mM Tris-HCl [pH 8.0], 150 mM NaCl, 0.05% Tween 20) containing 8% skim milk for 2 hours. The PVDF membrane was then incubated with anti-His antibody (1:3000) (TIANGEN) at room temperature for 2 hours. After the PVDF membrane was washed twice with TBST buffer, it was incubated with horseradish-peroxidase-conjugated secondary antibody (1:5000) (TransGen, Beijing, China) at room temperature for 2 hours. The PVDF membranes were exposed using enhanced chemiluminescence reagents (EasySee Western Blot Kit, TransGen, Beijing, China).

### Construction of the Δ*csgF*-*E*. *coli* K-12 BW25113 strain and mutagenesis

The linear fragments were amplified by PCR using primers P1 (5′- TTAAG TACGG GCGAT TTGGC GCATG ATGAA TTCTA AATAA AAAAT TGTTC GGAGG CTGCA GTGTA GGCTG GAGCT GCT -3′) and P2 (5′- ACATG ACGGC AACCA AAAGA AATAA GCGCT GCATG ATTAT TTTCC TTATG AAGCT GGGGC CATAT GAATA TCCTC CTTAG TTCCT ATTCC-3′) with 60-nt extensions that are homologous to regions adjacent to the *csgF* gene using the plasmid pKD3 as template. PCR fragments were subsequently transformed by electroporation into the *E*. *coli* K-12 strain BW25113—which carries the pKD46 plasmid—to obtain *csgF*::*Cm*. Mutants were colony purified once nonselectively at 37°C to eliminate pKD46. The *Cm* cassette was then removed by introducing the helper plasmid pCP20 into the mutant *csgF*::*Cm* and colony purified once nonselectively at 43°C. All *csgF* mutants were generated by using standard overlap PCR.

### Complementary assay and CR staining

The WT- and the Δ*csgF*-*E*. *coli* K-12 strain BW25113 were cultured in LB at 37°C until O.D._600_ reached 1.0. To induce curli production, 3 μL bacterial cultures were dropped on YESCA plates (10 g/L casamino acids, 1 g/L yeast extract, and 20 g/L bacto agar) supplemented with 50 μg/mL CR and 0.01 mM L-arabinose. Expression of CsgF or its mutants under control by the *ara* promoter in pBAD22 was induced by L-arabinose. The plates were placed at 26°C for 60 hours, and pictures were taken every 12 hours. *E*. *coli* that produced curli stained red, whereas curli-defective *E*. *coli* remained white. The experiments were performed in duplicate and repeated 3 times. The results are representative of replicates.

### Immunofluorescence assay

To carry out immunofluorescence assay, coding sequence of the full-length CsgF with a C-terminal 6×His tag was cloned into the pBAD22 vector. The resulted plasmid or a vacant pBAD22 vector was then transformed into either the WT- or the Δ*csgG*-*E*. *coli* strains. For each sample, 1 mL *E*. *coli* cell culture (O.D._600_ = 1.0) was loaded into a 1.5-mL Eppendorf tube. After removing the supernatant by centrifugation, each cell pellet was washed twice with 1×PBS. The cell pellet for each sample was then resuspended with 1 mL 1×PBS, and 200-μL suspension for each sample was transferred into the glass-bottom cell culture dish (NEST, ø15 mm) that was pretreated with 0.01% poly-L-lysine. Following incubation for 5 minutes, the redundant suspension was discarded. The bacteria attached to the dish were fixed by addition of 200 μL 4% (w/v) paraformaldehyde in 1×PBS for 2 hours, followed by washing twice with 1 mL 1×PBS for each sample.

The fixed bacteria were then blocked in 10% normal goat serum in 1×PBS for 30 minutes. After being blocked, the samples were incubated with primary antibody (mouse anti-His, 1:100, CMCTAG) for 2 hours, and then washed with 1×PBS 3 times, 5 minutes each. The samples were then stained with Alexa Fluor 546-conjugated secondary antibody (goat anti-mouse, 1:200, Invitrogen) for 1 hour. After being washed 3 times with PBS, they were mounted in Moviol Mounting Media with 4′,6-diamidino-2-phenylindole (DAPI; blue-fluorescing), a membrane-penetrating fluorescent dye that binds strongly to adenine-thymine rich regions in DNA. The bacteria images were acquired on the Delta Vision OMX V3 image system (GE Healthcare) with a ×100/1.40 NA oil-immersion objective lens (Olympus UPlanSApo) and a camera (Evolve 512 × 512, Photometrics). Images were processed and analyzed using Image J software (NIH). The experiments were performed in duplicate and repeated 3 times. The results are representative of replicates.

### Assay for peptide inhibition on curli production

Peptides G20-D53_CsgF, G20-N36_CsgF, G20-R36_CsgF, and A23-D46_CsgB were synthesized by GenScript. The WT- or the Δ*csgF*-*E*. *coli* K-12 strain BW25113 WT were cultured in LB at 37°C until O.D._600_ reached 1.0; 3 μL bacteria were dropped on the YESCA plates including 50 μg/mL CR and indicated concentrations of peptides. The plates were placed at 26°C for 60 hours, and pictures were taken every 12 hours. The experiments were performed in duplicate and repeated at least 3 times. The results are representative of replicates.

### Bacterial growth assay

The WT- and the Δ*csgF*-*E*. *coli* K-12 strain BW25113 were cultured in YESCA medium (10 g/L casamino acids, 1 g/L yeast extract) at 26°C until O.D._600_ reached 0.6. The peptide powder (G20-D53_CsgF, G20-N36_CsgF, or G20-R36_CsgF) was dissolved in dimethyl sulfoxide (DMSO) to a final concentration of approximately 1 mM; 1 μL peptide or DMSO (as control) was added into 1 mL culture. The final concentration of peptides in the cultures is 1 μM. The cultures were incubated at 26°C for 30 hours with a shaking rate of 200 rpm. The absorbance of each culture at 600 nm was measured every 3 hours over 30 hours using Ultrospec 10 (Biochrom US). The experiments were performed in triplicate and repeated 3 times.

## Supporting information

S1 Raw images for gels and blots Raw uncropped images of SDS-PAGE gels of Fig 2C (upper panel) and S3B Fig and western blot membranes of Fig 2C (bottom panel).
(PDF)

S1 Fig Workflow of cryo-EM structure determination of the CsgG-CsgF complex. (A) A representative raw image showing the CsgG-CsgF complex on the grid. (B) 2D class averages. (C) Workflow of data processing. (D) Particle orientation distribution of 3D reconstruction. (E) Gold-standard FSC curves. (F) Local resolution of final reconstruction of the CsgG-CsgF complex.
(TIF)

S2 Fig The NMR structure of CsgF and secondary structures of CsgF. (A) The NMR structure of the isolated full-length CsgF in ribbon representation. Secondary structures of residues 20–53 and residues 54–138 of CsgF are colored in violet and green, respectively. (B) Sequence alignment of CsgF from different bacterial strains. Symbols of secondary structures of CsgF are placed at the top of the aligned sequences: predicted secondary structures (light grey), cryo-EM structure (red), and NMR structure (cyan). The sequences alignment file can be found in S1 Data. Ec, *Escherichia coli*; Kp, *Klebsiella pneumonia*; Pr, *Pseudomonas resinovorans*; Sf, *Shigella flexneri*; St, *Salmonella typhimurium*.
(TIF)

S3 Fig Characterization of the CsgG-CsgF_20–59_ complex. (A) Gel filtration profiles of the full-length CsgG-CsgF complex (blue line) and the chymotrypsin-digested sample (red line) on a Superose 6 10/300 GL column. The full-length CsgG-CsgF complex has 2 elution peaks: P1 and P2. The chymotrypsin-digested CsgG-CsgF complex only has one major elution peak P3. Peak P3 corresponds to an apparently molecular mass of approximately 270 kDa. (B) 12% SDS-PAGE analysis of the gel filtration elution peaks P1, P2, and P3. Both CsgG and the CsgG-CsgF complex formed stable oligomers that were dissociated into monomers when the protein samples were heated at 95°C for 10 minutes. (C) Mass spectrometry analysis of the chymotrypsin-digested CsgG-CsgF complex to confirm that the digested sample containing the presence of the N-terminus of CsgF (residues 20–59). The observed molecular mass on the mass spectrum matches well with the calculated molecular mass of CsgF_20–60_.
(TIF)

S4 Fig Workflow of cryo-EM structure determination of the CsgG-CsgF_20–59_ complex. (A) A representative raw image showing that the CsgG-CsgF_20–59_ form filaments with different lengths on the grid. (B) 2D class averages. (C) Workflow of data processing. (D) Particle orientation distribution of 3D reconstruction. (E) Gold-standard FSC curves. (F) Local resolution of final reconstruction of the CsgG-CsgF_20–53_ complex and the CsgG-CsgF_20–59_ complex. The structures of the CsgG-CsgF_20–53_ complex and the CsgG-CsgF_20–59_ complex are shown in cartoon. CsgG and CsgF are colored in blue and violet, respectively. Residues 43–59 of CsgF in the CsgG-CsgF_20–59_ complex are highlighted in blue.
(TIF)

S5 Fig Analysis of packing modes in the filaments formed by the CsgG-CsgF_20–59_ complex. (A) Inter-complex homodimeric head-to-head packing mode (left). In this packing model, the last 6 residues of the CsgF_20–59_ fragment in both nonamers are invisible. The homodimeric inter-complex interaction is mediated by charge-charge interaction (middle and right). (B) Inter-complex heterodimeric head-to-tail packing mode (left). There exist 2 types of complexes: the CsgG-CsgF_20–59_ complex and the CsgG-CsgF_20–53_ complex. The heterodimeric inter-complex interaction is mediated by charge-charge interaction (middle and right). (C) In head-to-tail packing mode, the last 6 residues of the CsgF_20–59_ fragment in one of the complexes have weak densities. (D) Increased salt concentration (NaCl) in the sample buffer from 150 mM (left) to 300 mM (right) decreased lengths of filaments on grid as shown by representative images.
(TIF)

S6 Fig Effects of the CsgF-derived shorter peptides on curli production. Effects of peptides G20-F31_CsgF (left) and G20-R27_CsgF (right) on curli production to both the WT- and the Δ*csgF*-*E*. *coli* strains. Neither G20-F31_CsgF nor G20-R27_CsgF affected curli production to either the WT- or Δ*csgF*-*E*. *coli* K-12 BW25113 strains over an incubation time of 48 hours.
(TIF)

S1 Table Cryo-EM data collection, refinement, and validation statistics. (DOCX)

S1 Data Spreadsheet containing individual sheets for the underlying numerical data for Fig 2B, Fig 4D and sequences alignment file for S2B Fig. (XLSX)

## Abbreviations

3D: three-dimensional
CR: Congo red
cryo-EM: cryogenic electron microscopy
csg: curli-specific gene
DAPI: 4′,6-diamidino-2-phenylindole
DMSO: dimethyl sulfoxide
FSC: Fourier shell correction
GST: glutathione s-transferase
IPTG: isopropyl β-d-1-thiogalactopyranoside
ITC: isothermal titration calorimetry
NMR: nuclear magnetic resonance
O.D.: optical density
OM: outer membrane
PDB: Protein Data Bank
RMSD: root-mean-square deviation
WT: wild-type
