# Supplementary material for: Cryo-EM structure of the nonameric CsgG-CsgF complex and its implications for controlling curli biogenesis in Enterobacteriaceae
Source: PLoS Biol. 2020 Jun 19;18(6):e3000748. doi: 10.1371/journal.pbio.3000748 (PMC7304575; doi:10.1371/journal.pbio.3000748)
Supplement: S1 Raw images for gels and blots — Raw uncropped images of SDS-PAGE gels of Fig 2C (upper panel) and S3B Fig and western blot membranes of Fig 2C (bottom panel). (PDF) [file pbio.3000748.s001.pdf]

Fig 2C upper panel. GST pull-down experiments

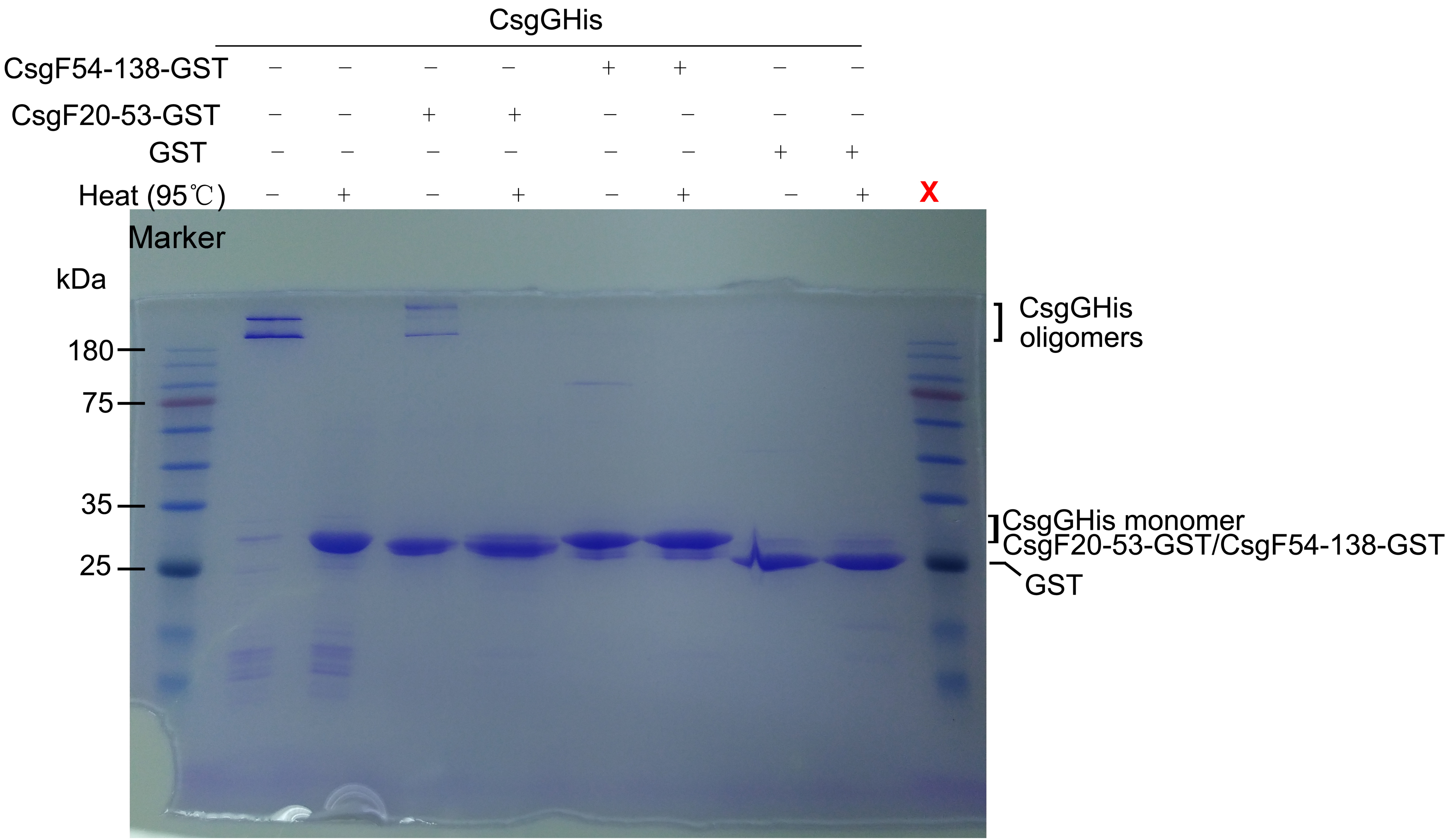

Image captured by camera.

Fig 2C bottom panel .GST pull-down experiments Western blot to detect CsgGHis

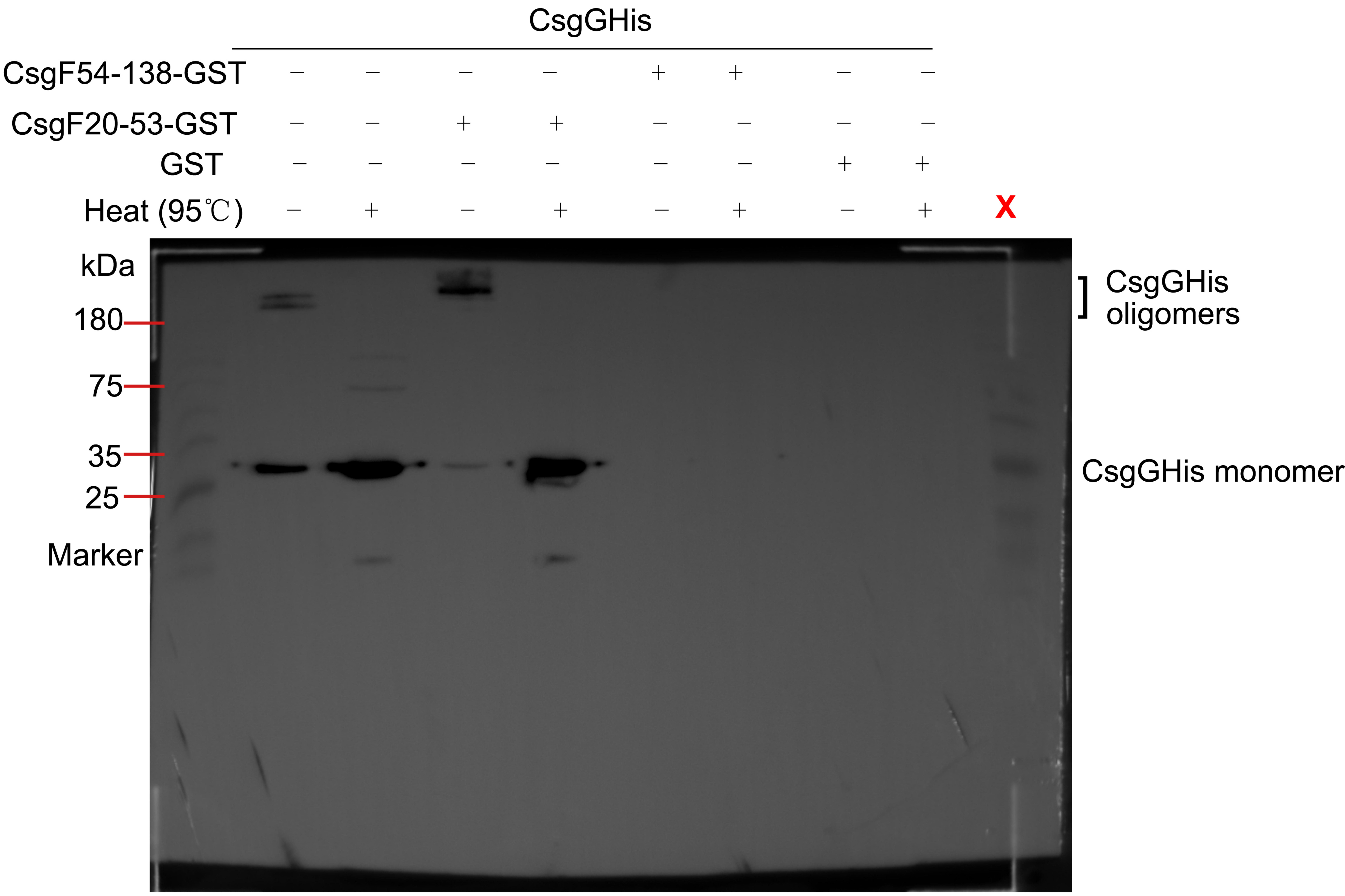

Image captured by ChemiScope3500 Mini (ClinX).

Fig S3B. 12% SDS-PAGE analysis of the gel filtration elution peaks P1, P2 and P3

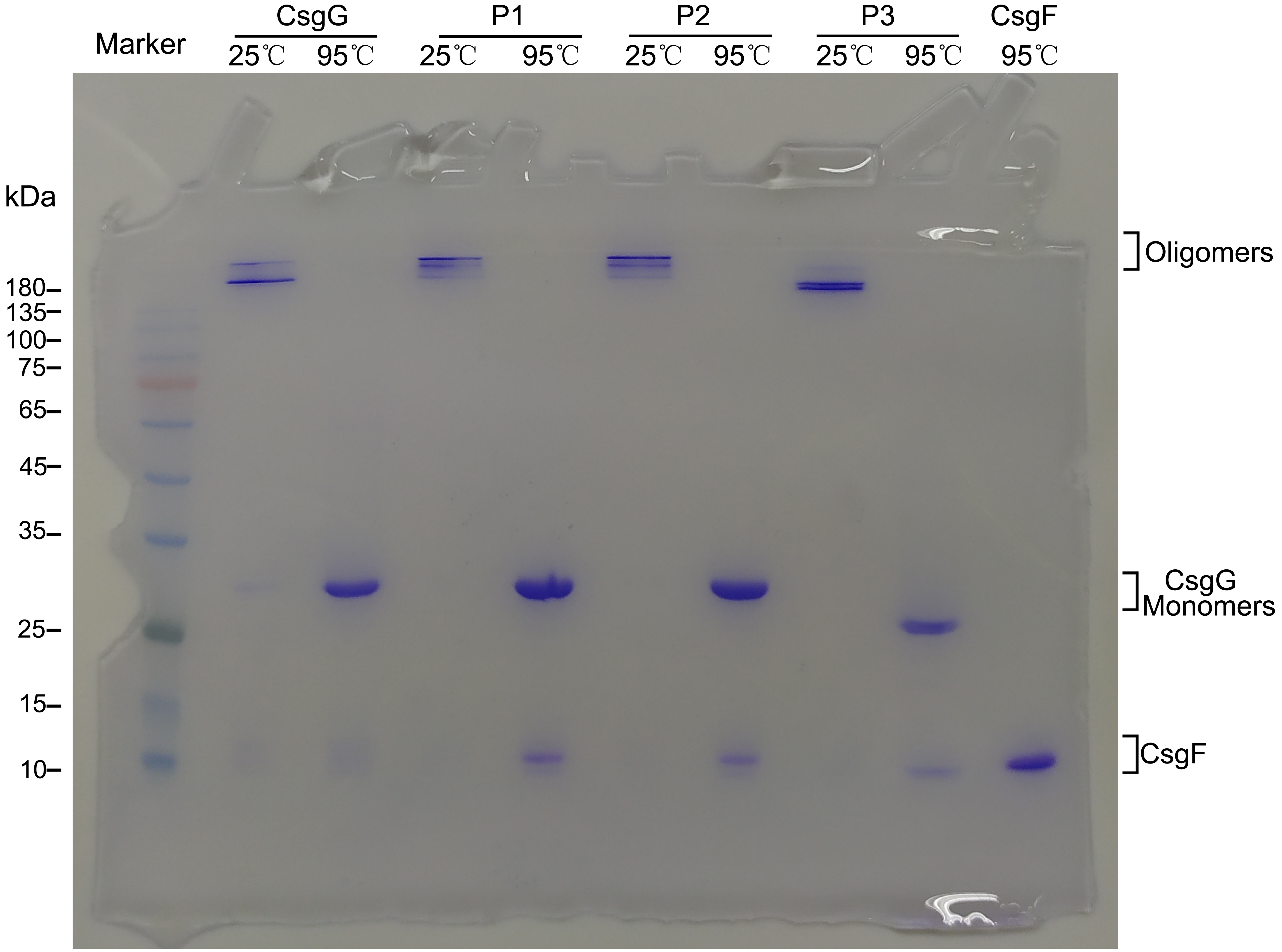

Image captured by camera.
