# Supplementary figures and images for: Cryo-EM structure of the nonameric CsgG-CsgF complex and its implications for controlling curli biogenesis in Enterobacteriaceae
Source: PLoS Biol. 2020 Jun 19;18(6):e3000748. doi: 10.1371/journal.pbio.3000748 (PMC7304575; doi:10.1371/journal.pbio.3000748)

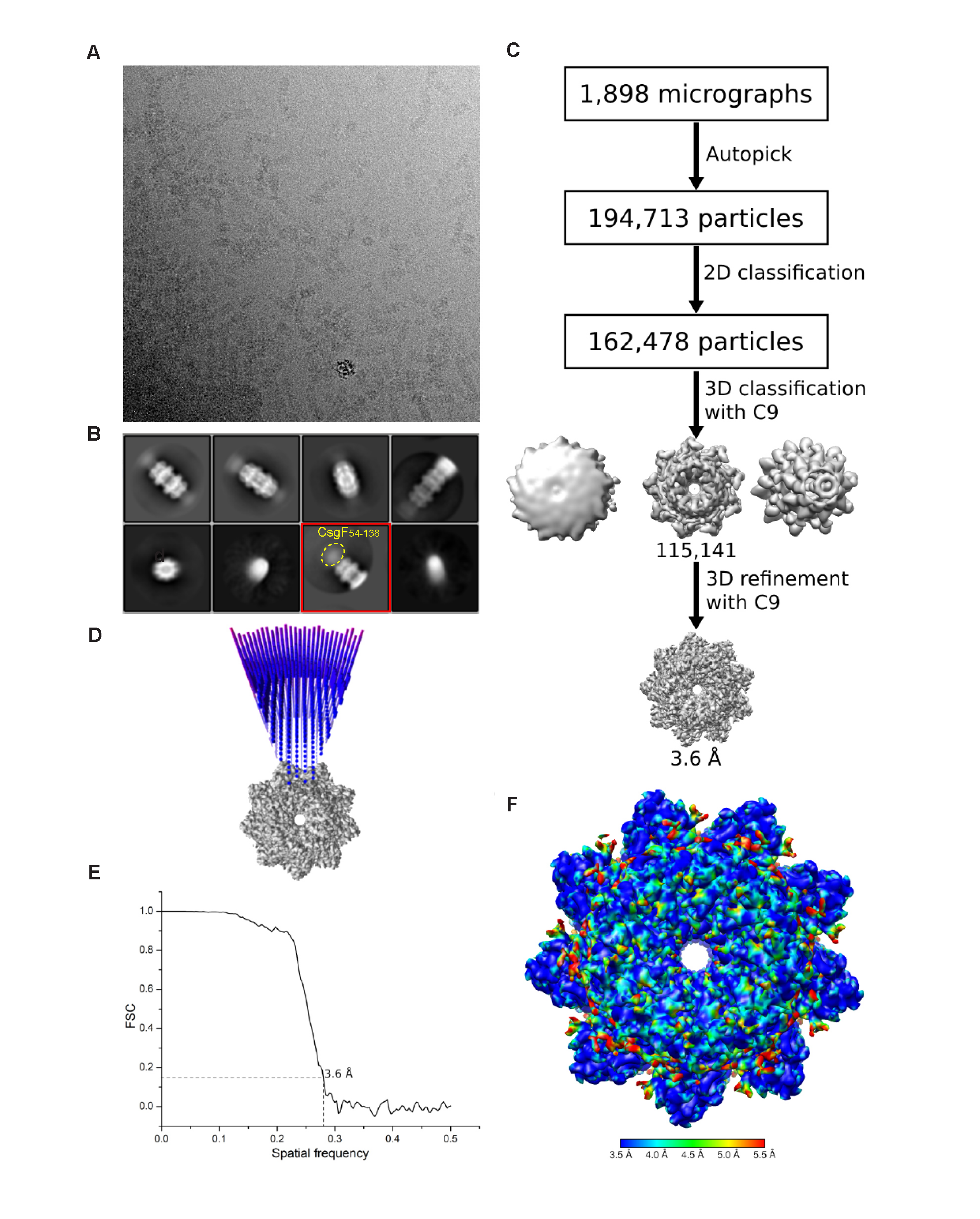

Supplement: S1 Fig — (A) A representative raw image showing the CsgG-CsgF complex on the grid. (B) 2D class averages. (C) Workflow of data processing. (D) Particle orientation distribution of 3D reconstruction. (E) Gold-standard FSC curves. (F) Local resolution of final reconstruction of the CsgG-CsgF complex. (TIF) [file pbio.3000748.s002.tif]

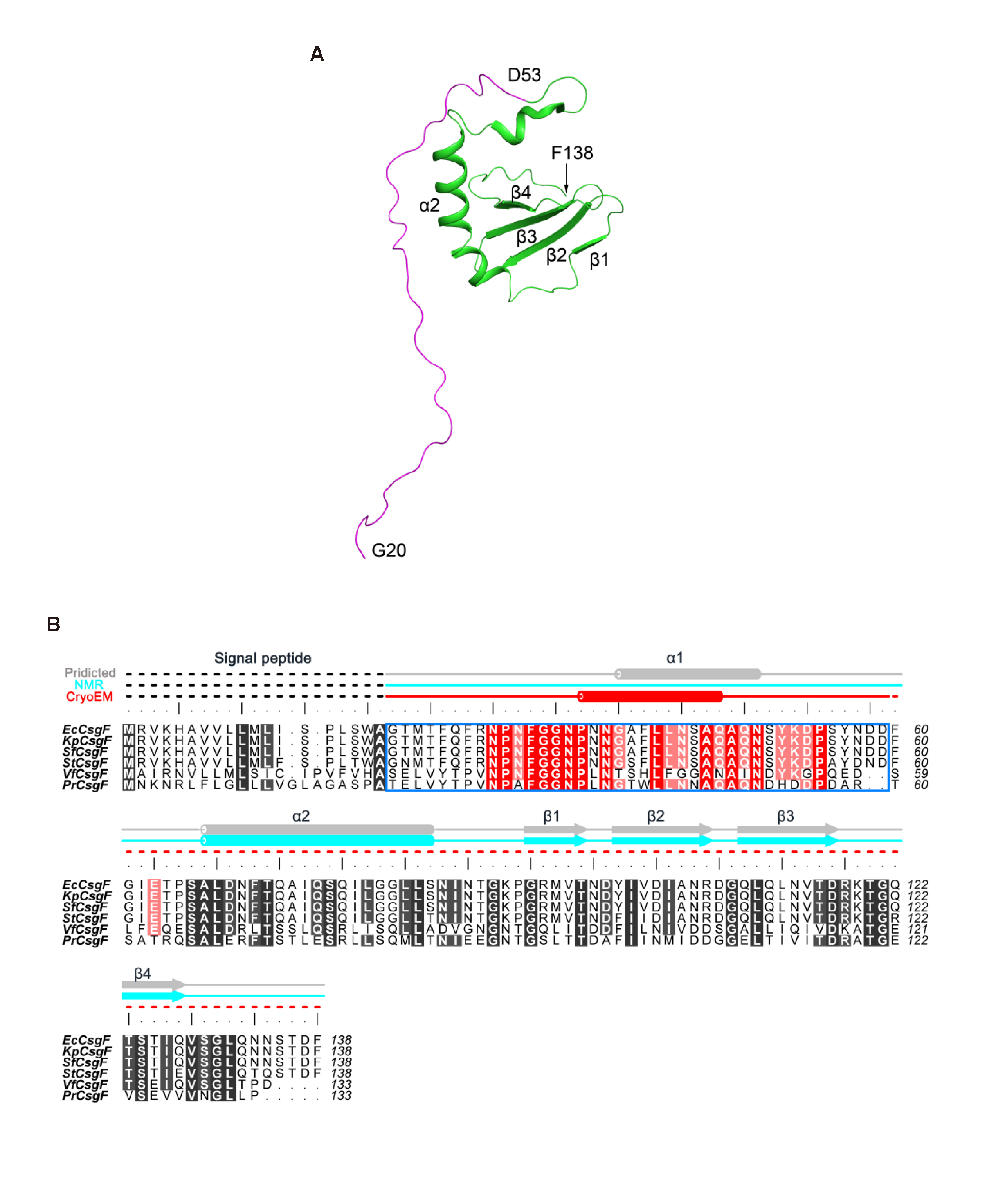

Supplement: S2 Fig — (A) The NMR structure of the isolated full-length CsgF in ribbon representation. Secondary structures of residues 20–53 and residues 54–138 of CsgF are colored in violet and green, respectively. (B) Sequence alignment of CsgF from different bacterial strains. Symbols of secondary structures of CsgF are placed at the top of the aligned sequences: predicted secondary structures (light grey), cryo-EM structure (red), and NMR structure (cyan). The sequences alignment file can be found in S1 Data. Ec, Escherichia coli; Kp, Klebsiella pneumonia; Pr, Pseudomonas resinovorans; Sf, Shigella flexneri; St, Salmonella typhimurium. (TIF) [file pbio.3000748.s003.tif]

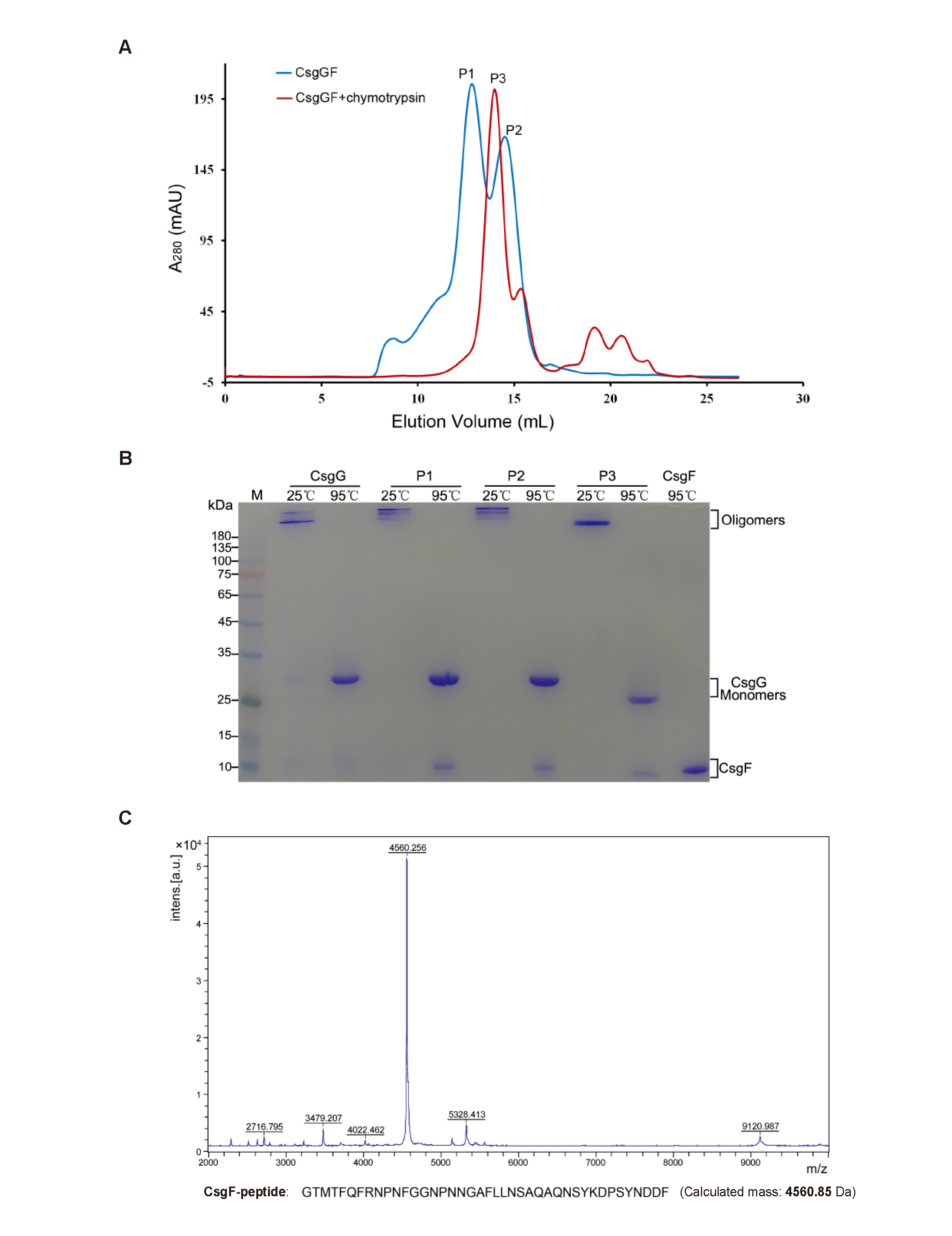

Supplement: S3 Fig — (A) Gel filtration profiles of the full-length CsgG-CsgF complex (blue line) and the chymotrypsin-digested sample (red line) on a Superose 6 10/300 GL column. The full-length CsgG-CsgF complex has 2 elution peaks: P1 and P2. The chymotrypsin-digested CsgG-CsgF complex only has one major elution peak P3. Peak P3 corresponds to an apparently molecular mass of approximately 270 kDa. (B) 12% SDS-PAGE analysis of the gel filtration elution peaks P1, P2, and P3. Both CsgG and the CsgG-CsgF complex formed stable oligomers that were dissociated into monomers when the protein samples were heated at 95°C for 10 minutes. (C) Mass spectrometry analysis of the chymotrypsin-digested CsgG-CsgF complex to confirm that the digested sample containing the presence of the N-terminus of CsgF (residues 20–59). The observed molecular mass on the mass spectrum matches well with the calculated molecular mass of CsgF20–60. (TIF) [file pbio.3000748.s004.tif]

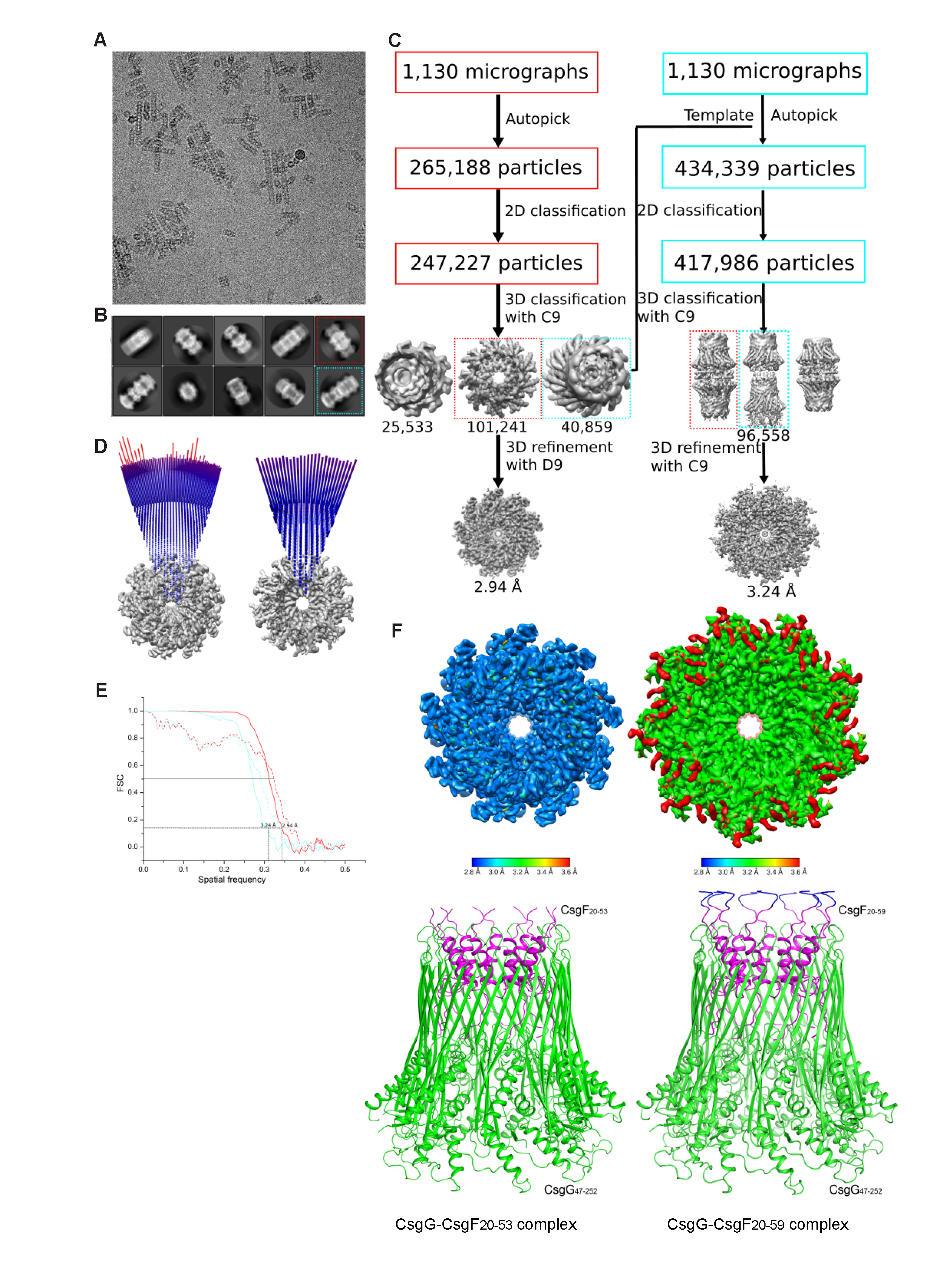

Supplement: S4 Fig — (A) A representative raw image showing that the CsgG-CsgF20–59 form filaments with different lengths on the grid. (B) 2D class averages. (C) Workflow of data processing. (D) Particle orientation distribution of 3D reconstruction. (E) Gold-standard FSC curves. (F) Local resolution of final reconstruction of the CsgG-CsgF20–53 complex and the CsgG-CsgF20–59 complex. The structures of the CsgG-CsgF20–53 complex and the CsgG-CsgF20–59 complex are shown in cartoon. CsgG and CsgF are colored in blue and violet, respectively. Residues 43–59 of CsgF in the CsgG-CsgF20–59 complex are highlighted in blue. (TIF) [file pbio.3000748.s005.tif]

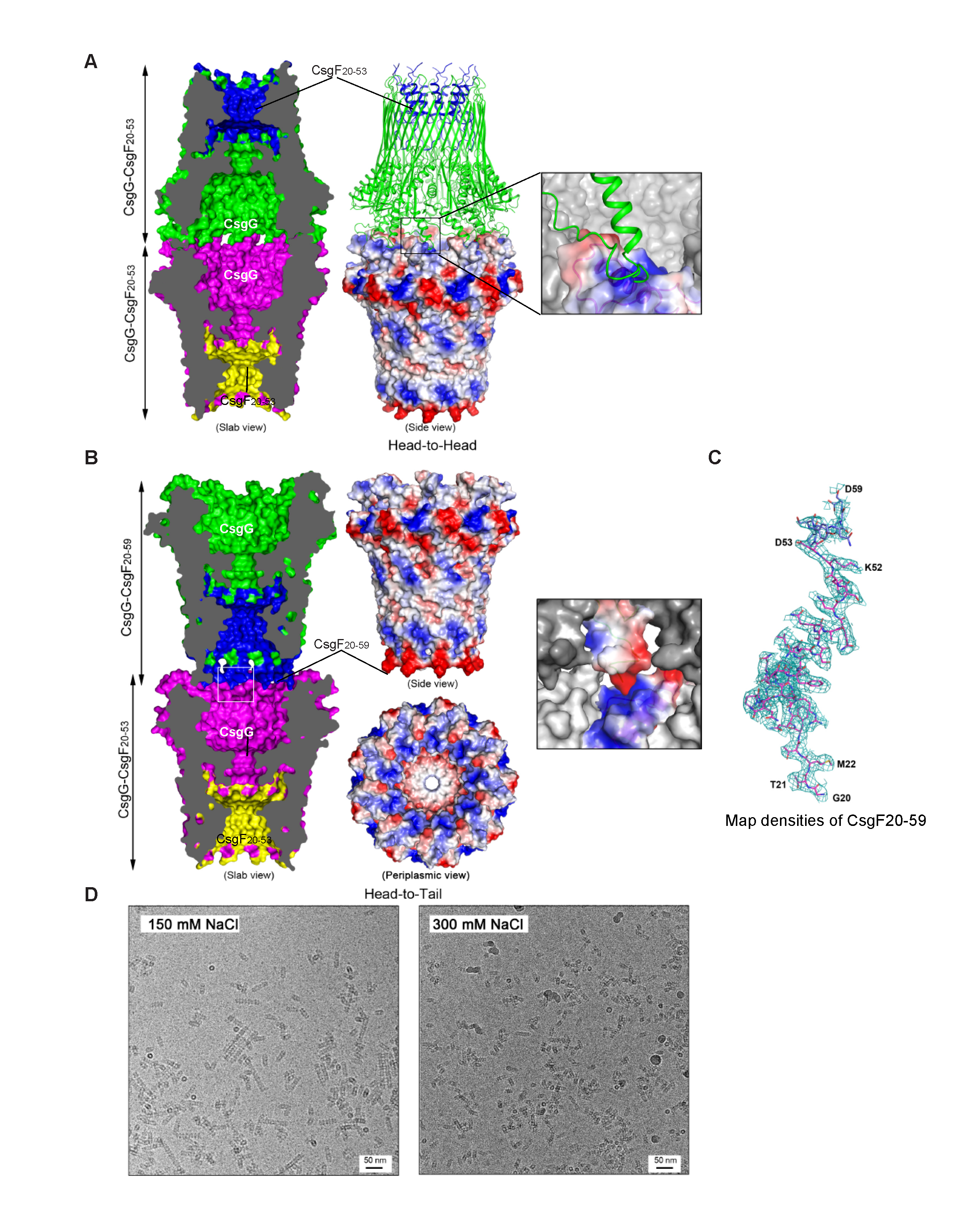

Supplement: S5 Fig — (A) Inter-complex homodimeric head-to-head packing mode (left). In this packing model, the last 6 residues of the CsgF20–59 fragment in both nonamers are invisible. The homodimeric inter-complex interaction is mediated by charge-charge interaction (middle and right). (B) Inter-complex heterodimeric head-to-tail packing mode (left). There exist 2 types of complexes: the CsgG-CsgF20–59 complex and the CsgG-CsgF20–53 complex. The heterodimeric inter-complex interaction is mediated by charge-charge interaction (middle and right). (C) In head-to-tail packing mode, the last 6 residues of the CsgF20–59 fragment in one of the complexes have weak densities. (D) Increased salt concentration (NaCl) in the sample buffer from 150 mM (left) to 300 mM (right) decreased lengths of filaments on grid as shown by representative images. (TIF) [file pbio.3000748.s006.tif]

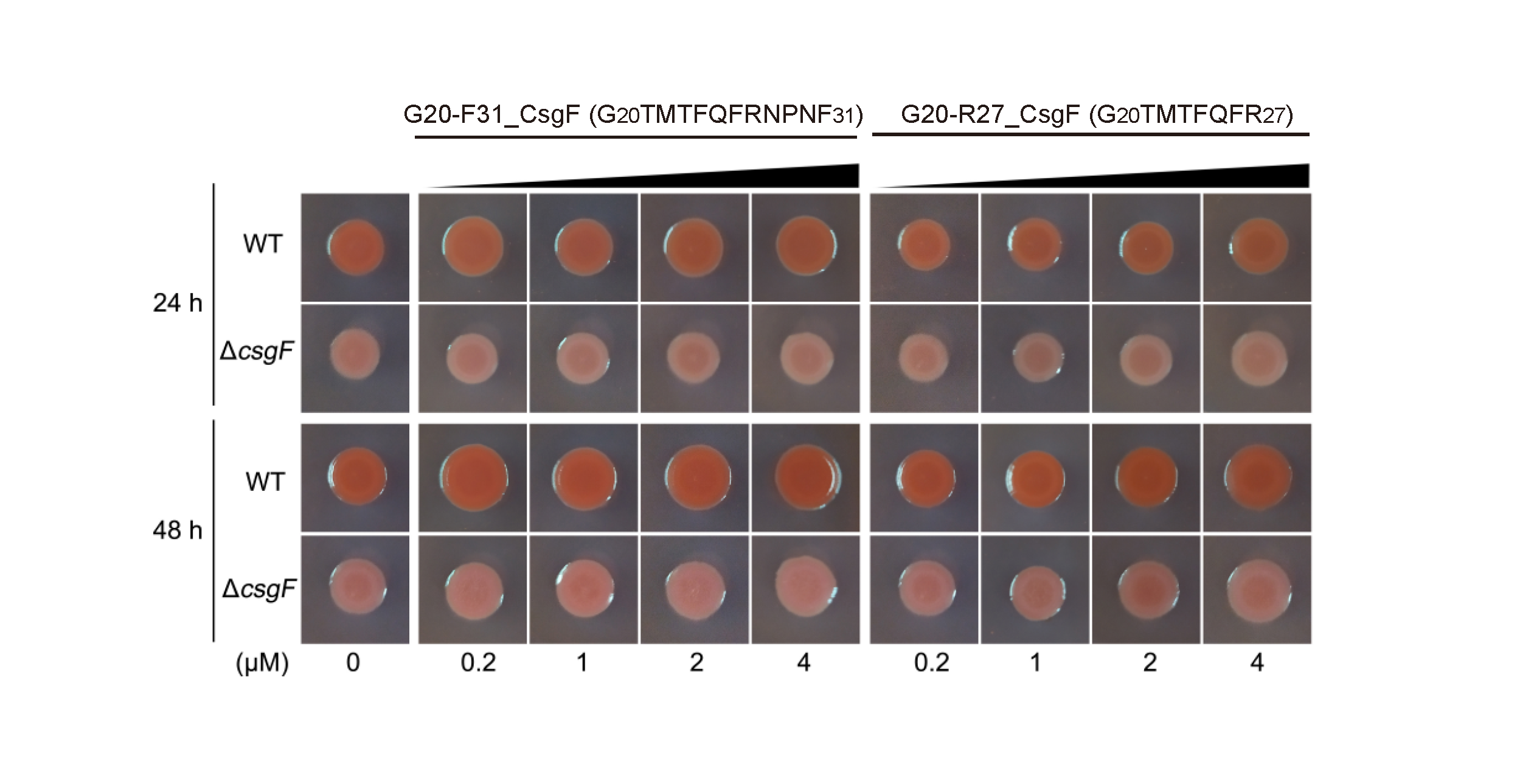

Supplement: S6 Fig — Effects of peptides G20-F31_CsgF (left) and G20-R27_CsgF (right) on curli production to both the WT- and the ΔcsgF-E. coli strains. Neither G20-F31_CsgF nor G20-R27_CsgF affected curli production to either the WT- or ΔcsgF-E. coli K-12 BW25113 strains over an incubation time of 48 hours. (TIF) [file pbio.3000748.s007.tif]
