# Supplementary material for: Cryo-EM structure of the nonameric CsgG-CsgF complex and its implications for controlling curli biogenesis in Enterobacteriaceae
Source: PLoS Biol. 2020 Jun 19;18(6):e3000748. doi: 10.1371/journal.pbio.3000748 (PMC7304575; doi:10.1371/journal.pbio.3000748)
Supplement: S1 Table — (DOCX) [file pbio.3000748.s008.docx]

**S1 Table**. **Cryo-EM data collection, refinement, and validation statistics.**

|  | CsgG-CsgF | CsgGF_20-53_ | CsgGF_20-59_ |
| --- | --- | --- | --- |
| **Data collection and processing** |  |  |  |
| Magnification | 130,000 | 130,000 | 130,000 |
| Voltage (kV) | 200 | 200 | 200 |
| Electron exposure (e^–^/Å^2^) | 50 | 48 | 48 |
| Defocus range (μm) | 1~2.5 | 1~2.5 | 1~2.5 |
| Pixel size (Å) | 1 | 1 | 1 |
| Symmetry imposed | C9 | D9 | C9 |
| Initial particle images (no.) | 194,713 | 265,188 | 434,339 |
| Final particle images (no.) | 115,141 | 101,241 | 96,558 |
| Map resolution (Å) | 3.60 | 2.94 | 3.24 |
| FSC threshold | 0.143 | 0.143 | 0.143 |
| Map resolution range (Å) | 3.6~6.0 | 2.9~3.2 | 3.2~5.5 |
| **Refinement** |  |  |  |
| Initial model used (PDB code) | 3X2R | 3X2R | 3X2R |
| Model resolution (Å) | 3.60 | 2.94 | 3.24 |
| FSC threshold | 0.5 | 0.5 | 0.5 |
| Model resolution range (Å) | 3.6~6.0 | 2.9~3.2 | 3.2~5.5 |
| Map sharpening *B* factor (Å^2^) | -188.333 | -133.503 | -157.95 |
| Model composition |  |  |  |
| Non-hydrogen atoms | 20,277 | 16,623 | 17,100 |
| Protein residues | 2,619 | 2,160 | 2,214 |
| Ligands | - | - | - |
| *B*-factors(Å^2^) |  |  |  |
| Protein | 42.48 | 43.93 | 26.23 |
| Ligand | - | - | - |
| R.m.s. deviations |  |  |  |
| Bond lengths (Å) | 0.010 | 0.009 | 0.006 |
| Bond angles (º) | 0.862 | 1.043 | 0.970 |
| Validation |  |  |  |
| MolProbity score | 2.41 | 2.42 | 1.99 |
| Clashscore | 16.16 | 5.94 | 4.81 |
| Poor rotamers (%) | 1.23 | 6.03 | 1.94 |
| Ramachandran Plot |  |  |  |
| Favored (%) | 88.15 | 91.53 | 90.96 |
| Allowed (%) | 11.85 | 8.47 | 9.04 |
| Disallowed (%) | 0 | 0 | 0 |
